# Sintering Method-Dependent Hydroxyapatite Coatings Drive Enhanced Gingival Fibroblast Behavior on Titanium Implant Surfaces

**DOI:** 10.3390/ma19122573

**Published:** 2026-06-15

**Authors:** Andreia Bandeira Luís, Narayan Sahoo, Beatriz Ferreira Fernandes, António Mata, Óscar Carvalho, Joana Faria Marques

**Affiliations:** 1 CEMBDE-Cochrane Portugal, Faculdade de Medicina Dentária, Universidade de Lisboa, 1600-277 Lisbon, Portugal; andreia.vieira@edu.ulisboa.pt (A.B.L.); admata2@edu.ulisboa.pt (A.M.); jmarques2@edu.ulisboa.pt (J.F.M.); 2 GIBBO-UICOB, Faculdade de Medicina Dentária, Universidade de Lisboa, 1600-277 Lisbon, Portugal; beatriz-ferreira@edu.ulisboa.pt; 3 Center for MicroElectroMechanical Systems (CMEMS-UMinho), Universidade do Minho, 4800-058 Guimarães, Portugal; ilovsai@gmail.com; 4 LABBELS Associate Laboratory, University of Minho, 4710-057 Braga, Portugal; 5 LIBPhys-UL, Faculdade de Medicina Dentária, Universidade de Lisboa, 1600-277 Lisbon, Portugal

**Keywords:** titanium implants, hydroxyapatite, laser surface-texturing, laser sintering, biocompatibility

## Abstract

Implant surface optimization aims to reduce osteointegration process time and prevent failures. Here, we report a novel laser-assisted approach for incorporating hydroxyapatite into titanium implant surfaces and evaluate the resulting biological response. Titanium discs were fabricated by Nd:YVO4 laser texturing and coated with hydroxyapatite using either conventional or laser sintering, according to seven study groups: flat titanium (TiL), laser-textured titanium with 0.25 and 0.8 mm patterns (TiT025 and TiT08), and laser-textured titanium with 0.25 and 0.8 mm patterns plus bioactive coating using conventional sintering (TiT025CS and TiT08CS) or laser sintering (TiT025LS and TiT08LS). Human gingival fibroblasts (HGF hTERT) were cultured on discs to assess adhesion, morphology, viability, and cytokine secretion. Surface texturing alone did not significantly affect fibroblast viability over 7 days (*p* > 0.05). Hydroxyapatite coatings significantly reduced viability on both patterns when conventionally sintered (*p* < 0.05), whereas laser-sintered coatings did not cause a significant decrease; overall viability was higher in LS than in CS samples (*p* < 0.05). Scanning electron microscopy after 24 h showed adherent cells on all surfaces. IL-1β secretion was consistently lower than IL-10 secretion during the 3-day study period. When normalized to cell viability, these findings remained consistent. At day 1, IL-1β/viability and IL-10/viability ratios were similar across groups. By day 3, the IL-1β/viability ratio decreased in all groups, with TiT08 showing significantly lower values than TiT08CS (*p* < 0.05). In contrast, the IL-10/viability ratio increased in coated patterned samples (TiT025, TiT025CS, TiT025LS, TiT08, TiT08CS, and TiT08LS). In conclusion, the 0.25 mm laser-textured pattern combined with optimized hydroxyapatite sintering elicited a more favorable cytokine secretion profile compared to the 0.8 mm pattern, suggesting a reduced pro-inflammatory response.

## 1. Introduction

The osseointegration process is a coordinated and organized healing mechanism in which cells play a central role, with each cell having a specific task defined in time and space [1]. This process is essential for the formation of bone around the implant surface and for ensuring its stability, allowing it to be rehabilitated ([sposito, Ardebili [2]). However, in some cases, failures in this process occur, which may be caused by a decrease in the number or activity of osteogenic cells, an increase in osteoclast activity, an imbalance between anabolic and catabolic factors in bone formation, osteolysis due to mechanical stress, or insufficient or absent vascularization in the peri-implant tissues [1,3].

The success of the osseointegration process depends not only on the factors mentioned above but also on the administration of certain drugs, such as cyclosporine A, bisphosphonates, or simvastatin, which may inhibit bone formation [1,4]. Other factors, particularly those related to the implant—such as its design, material, chemical composition, dimensions, diameter, and surface treatment—also significantly affect the success rate of osseointegration [5,6]. Osseointegration and the biological processes associated with it remain an ongoing area of research. The primary goal is to enhance the predictability of this entire biological, cellular, and molecular process, thereby improving the post-operative experience for patients. A continuous concern is the reduction of exaggerated inflammatory responses, which can lead to implant failure or peri-implant complications [7,8,9,10].

Modifications to implant surfaces aim to increase the contact area with the bone by enhancing surface roughness, as previously discussed, improving cell adhesion, and stimulating the proliferation of extracellular matrix components, without promoting bacterial adhesion [1,11,12]. The goal is to create controlled surface conditions (topography, roughness, and wettability) that support optimal biological performance while minimizing the risk of microbial colonization [13]. For example, fibroblasts exhibit lower adhesion on rougher surfaces; therefore, smoother surfaces—Ra ≈ 0.08–1 μm—are associated with higher cell adhesion rates [14].

Several techniques have been proposed in the literature, some of which remove material from the implant surface—subtraction techniques—while others add new substances to the implant surface—addition or additive techniques [15,16].

Among these techniques, laser-based methods for implant surface texturing are particularly interesting, since they involve the removal of substrate material through laser ablation. Material removal quantity and quality, i.e., geometry/topography and cleanness, depend on pulse duration/pulse width of the laser beam and spot, but in all cases, surface ionization occurs, leading to changes in texture, morphology, and chemical composition [17,18].

Laser-induced texture changes allow for precise and controlled surface modifications ensuring a reproducible pattern with defined characteristics [19,20]. This is the only method that enables texture alterations without direct contact with the implant surface, thus preventing surface contamination [19,21]. On the other hand, chemical agents, such as hydroxyapatite, can be added to the implant surface, promoting biofunctionalization. Incorporating synthetic hydroxyapatite into the implant surface is a promising modification strategy due to its chemical similarity to bone, promoting favorable biological responses. This includes enhanced cell adhesion via increased fibronectin and vitronectin adsorption, as well as osseoconduction, marked by increased osteoblast recruitment [22,23,24,25].

However, challenges exist in incorporating hydroxyapatite, such as the need to create a coating that ensures long-lasting and stable adhesion, forming a homogeneous surface with uniform material distribution. Another significant challenge is achieving a strong bond between hydroxyapatite and titanium, minimizing mechanical differences between the two materials, in order to reduce stress at the interface and to prevent fractures or delamination [26,27,28].

As a result, the addition of bioactive substances such as hydroxyapatite to the implant surface must be stabilized to optimize its physical and chemical properties. Stabilization can be achieved through sintering processes, which involve a thermal procedure that fuses fine particles of a material into a solid mass using heat and/or pressure, without fully melting the particles. This process can be carried out conventionally or via laser [6]. Conventional sintering is a simple technique in which the bioactive material is prepared at room temperature and then heated to temperatures ranging from 950° to 1175° for 1 h in an oven or similar apparatus, without any increase in pressure. While simple and low in cost, this process is time-consuming and energy-intensive [26,27,29].

Laser sintering, an additive technique, uses a laser (neodymium-doped yttrium aluminum garnet (Nd:YAG) laser or neodymium-doped yttrium orthovanadate (Nd:Yv04) or CO_2_ laser) as a heat source to sinter hydroxyapatite powder. This process is controlled by CorelDraw version 26.0, allowing the addition of the material layer by layer to create precise structures. In an in vitro study by J. Mesquita-Guimarães et al., no significant differences were found in the viability of an osteoblast cell line (MC3T3-E1) when exposed to titanium discs coated with hydroxyapatite sintered either conventionally or by CO_2_ laser [30].

The aim of this study was to determine how texturing of titanium implant surfaces using laser techniques and subsequent biofunctionalization with hydroxyapatite using laser or conventional techniques influences the viability, morphology and inflammatory response of human gingival fibroblasts.

## 2. Methods and Materials

### 2.1. Sample Processing

Based on a titanium alloy bar (Ti-6Al-4V), with the chemical characteristics described in Table 1, discs of 8 mm in diameter and approximately 2.5 mm in height were obtained, which were subsequently polished with sandpaper to remove surface burs and debris that could interfere with the results. The production of samples was performed at the CMEMS facilities.

### 2.2. Texture Pattern

After producing the samples, surface texturing of titanium samples was carried out using a Nd:YV04 laser (OEM Plus, SISMA, Vincenza, Italy), operating at λ = 1064 nm and a pulse width of 10 ns and with an initial focus at 11.1 cm under normal atmospheric pressure and with constant air renewal using a fan and a jet of compressed air, promoting the removal of debris from the sample surface. Laser specifications are detailed in Table 2.

The pattern of the samples was a checkerboard formed by grooves and ridges. This pattern was drawn using CorelDraw software, v26.0 and later transferred to the laser. Two designs were created, differing in the length of the crest and groove: in the “08” groups, the length of the crest and groove was 0.8 mm, while in the “025” groups, the length was 0.25 mm. The two designs did not present differences in the distance between the ridges, but rather in the surface area of the non-textured material. After texturing, all samples were disinfected again in an isopropyl alcohol solution for 1 min in an ultrasound bath.

### 2.3. Bioactive Functionalization

The addition of hydroxyapatite to the samples was done using a suspension of hydroxyapatite powder (nanoXIM Hap400, Fluidinova, Maia, Portugal) and water with a concentration of 0.133 g/mL by dip coating. The chemical formula and other parameters are described in Table 3, according to the information provided by the manufacturer.

After the samples were coated with hydroxyapatite, they were sintered using one of two different methods: laser or conventional. Laser-sintered samples used a Carbon Dioxide Laser (BD-50C, Bende Machinery Co., Ltd., Chongqing, China) with a maximum power of 50 W and a fundamental wavelength λ = 1064 nm (complete specifications are detailed in Table 4). Conventional sintered samples were sintered in a furnace at a temperature of 950 to 1175 °C for 1 h.

Samples were allocated to one of seven study groups: polished titanium (TiL); texturized titanium in a 0.25 pattern (TiT025); texturized titanium in a 0.25 pattern plus a bioactive with conventional sintering (TiT025CS); texturized titanium in a 0.25 pattern plus a bioactive with laser sintering (TiT025LS); texturized titanium in a 0.8 pattern (TiT08); texturized titanium in a 0.8 pattern plus a bioactive with conventional sintering (TiT08CS); and texturized titanium in a 0.8 pattern plus a bioactive with laser sintering (TiT08LS).

### 2.4. Surface Characterization

#### 2.4.1. Surface Roughness Analysis

Surface roughness measurements were performed for all groups using a contact profilometer (SurfTest SJ-201, Mitutoyo, Tokyo, Japan) equipped with a sharp diamond stylus (2 μm tip diameter). Ten measurements were acquired at randomly selected areas of each sample, using a scanning speed of 0.5 mm/s and a sampling length of 0.8 mm. Profilometry was conducted perpendicular to the texture direction to ensure accurate surface characterization. The roughness parameter measured was Ra, defined as the arithmetic average roughness. Surface measurements were strictly restricted to the uncoated metallic substrates (TiL, TiT025, and TiT08). Since contact mechanical profilometry utilizes a sharp diamond stylus under physical load, executing these measurements on the thin, newly deposited hydroxyapatite coatings posed a severe risk of micro-fracturing, scratching, or delaminating the brittle ceramic overlayer. To preserve the structural topography of the biofunctionalized layers for subsequent biological assays, quantitative contact profilometry was omitted for the coated groups, and surface integrity was instead qualitatively monitored via scanning electron microscopy (SEM).

#### 2.4.2. Surface Wettability

The wettability of all treated samples—TiT, TiT025, TiT08, TiT025CS, TiT025LS, TiT08CS and TiT08LS groups—was evaluated by contact angle measurements using two probe liquids: deionized water (H_2_O) and diiodomethane (CH_2_I_2_). These probes were selected to provide polar and nonpolar contact angles for subsequent surface energy calculations.

Measurements were performed at room temperature using the sessile drop method with an optical goniometer (OCA 15 Plus, Dataphysics, Filderstadt, Germany). A 5 μL droplet of ultrapure deionized water (18.2 MΩ·cm) was dispensed from a micrometric syringe at a rate of 2.5 μL/s, brought into contact with the sample surface, and allowed to stabilize for 15 s before analysis. Five measurements were taken on each sample, and the mean value was recorded as the result. Prior to testing, all samples were ultrasonically cleaned in isopropyl alcohol for 1 min to remove surface contaminants.

### 2.5. Cell Culture

The immortalized human gingival fibroblasts were HGF hTERT-T0026 (Applied Biological Materials Inc., Richmond, BC, Canada). Cells were cultured in a 75 cm^3^ culture flask in an atmosphere of 5% CO_2_ and 100% humidity at 37.0 °C in a culture medium composed of Dulbecco’s Modified Eagle’s Medium—DMEM (Biowhittaker, Lonza, Basel, Switzerland)—supplemented with 10% fetal bovine serum (Biowest, Nuaillé, France) and 100 UmL^−1^ penicillin and 100 μg/mL streptomycin (Lonza ^TM^, Basel, Switzerland).

When cells reached 80% confluence they were detached using trypsin-EDTA (Lonza ^TM^, Basel, Switzerland), centrifuged at approximately 100× *g* for 5 min and re-suspended in culture media. Cells were seeded on the discs and distributed in 48-well plates. Sample discs were distributed in 48-well culture plates (Corning, NY, USA) under sterile conditions at a density of 1.0 × 10^4^ cells/well and cultured at 37 °C for all biological assays. All experiments were conducted using a fifth passage.

### 2.6. Cell Viability and Morphology Assays

Eight groups were considered (the seven described above and a positive control). This assay was evaluated in three independent experiments with duplicates of 5 samples per group (N = 15). Cell viability and proliferation were evaluated using a resazurin-based viability assay—cell-titer Blue^®^ reagent (Promega^®^, Madison, WI, USA)—according to the manufacturer’s protocol. The conversion rate was measured as fluorescence intensity in arbitrary fluorescence units (AU) after 1, 3 and 7 days of culture. Fluorescence intensity was detected at excitation/emission wavelengths of 560/590 nm using a fluorescence spectrometer, the Victor Nivo Multimode Plate Reader (PerkinElmer^®^, Waltham, MA, USA).

### 2.7. Cell Morphology

Fibroblasts were cultured on discs for 1 day. Culture cells were washed with phosphate-buffered saline (PBS—VWR^®^, Radnor, PA, USA) and then fixed with 2.5% glutaraldehyde solution (VWR^®^, Radnor, PA, USA) for 1 h. For the dehydration process, serial dilutions of ethanol were used. Samples were metallized using a gold target in a JEOL JFC 1200 (Jeol Ltd., Tokyo, Japan) sputtering chamber. Samples were observed under JEOL JSM5200-LV (Jeol Ltd., Tokyo, Japan) and secondary images were created at acceleration voltages of 15 kV and 25 kV with different magnification (100, 150, 180 and 200×). Two calibrated researchers performed the image analysis, considering cell morphology, spreading and adhesion to the materials.

### 2.8. Interleukine-1β and Interleukine-10 Quantification Assay

The quantification of IL-1β and IL-10 present on the supernatant was conducted after 1 and 3 days of cell culture, using a Human IL-1β or IL-10 kit (DuoSet ELISA (R&D Systems Inc., Minneapolis, MN, USA)) according to the manufacturer’s instructions.

After the incubation period, the samples were read on a Victor Nivo Multimode Plate Reader (PerkinElmer^®^, EUA) at λ = 540 nm and λ = 450 nm.

The results were obtained in absorbance units (nm) and subsequently converted into pg/mL using the linear regression function of the absorbance values recorded by the calibration line. One experiment with duplicates of 3 samples per group was performed (*n* = 6).

### 2.9. Statistical Analysis

Statistical analyses were performed using IBM^®^ SPSS^®^ 29.0 statistics software for Mac (SPSS, Chicago, IL, USA). The Shapiro–Wilk test was used to test data for normality. For cell viability and interleukin quantification data the comparisons between groups were carried out using two-way ANOVA, and significant differences between groups were identified with Tukey’s post hoc test. The significance level was set as *p* < 0.05. All data are presented as the mean ± standard deviation (SD).

## 3. Results

### 3.1. Surface Characterization

#### Surface Roughness and Wettability

Surface roughness was observed among the groups TIL, TiT025 and TiT08, as detailed in Table 5. The table also depicts the wettability values for all the groups under study.

### 3.2. Cell Viability

**Figure 1:**
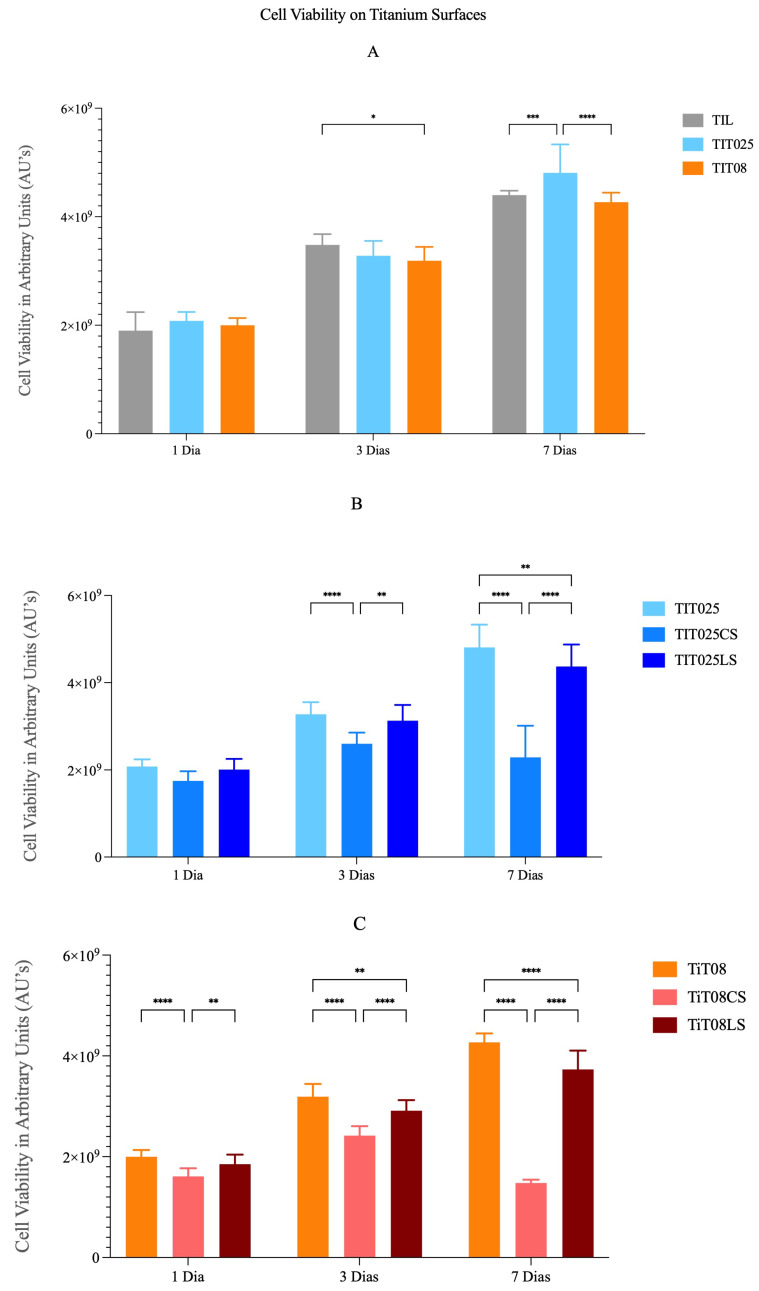
Bar charts depicting human gingival fibroblast (HGF) cell viability after 24 h of culture on the engineered titanium surfaces. (**A**) HGF viability on laser-textured metallic substrates (TiT025 and TiT08) compared to the smooth titanium control (TiL); (**B**) HGF viability on the 0.25 mm patterns modified with conventionally sintered (TiT025CS) or laser-sintered (TiT025LS) hydroxyapatite (HA) coatings; (**C**) HGF viability on the 0.8 mm patterns modified with conventionally sintered (TiT08CS) or laser-sintered (TiT08LS) HA coatings. Cell viability is expressed in arbitrary units (AUs). Data are presented as mean ± standard deviation (SD) from three independent experiments (*n* = 15 per group). Statistical significance: * *p* < 0.05, ** *p* < 0.01, *** *p* < 0.005, **** *p* < 0.001.

Overall, the addition of hydroxyapatite as a bioactive reduced cell viability, independent of the sintering method and timepoint of viability measurement, as depicted in Figure 1. When evaluating the effect of texturing the implant surface, as shown in Figure 1A (smooth titanium vs. the two texturing patterns), it was observed that there were no statistically significant differences (*p* > 0.05) at day 1. At day 3, it was observed that the textured pattern showed less viability than smooth titanium, and that the decrease in viability was statistically significant for TiT08 (*p* < 0.05). At day 7, the results showed an increase in cell viability in TiT025, which was statistically significant for TiT08 (*p* < 0.05).

Regarding the influence of the bioactive on textured surfaces over the 7 days (Figure 1B,C), it is possible to observe that in the 0.25 and 0.8 patterns, there is a decrease in cell viability when a bioactive is added compared to the pattern without a bioactive. Considering pattern 0.25, in the conventional sintering group (TiT025CS) the decrease was significant at days 3 and 7 (*p* < 0.05); when the bioactive is added by a laser sintering technique (TiT025LS), this decrease in viability is only significant (*p* < 0.05) at day 7. Considering pattern 0.8, at day 1 the decrease in cell viability was statistically significant (*p* < 0.05) for the conventional sintering group (TiT08CS) (*p* < 0.05) and at days 3 and 7 the decrease in cell viability was statistically significant (*p* < 0.05) for both sintering methods.

**Figure 2:**
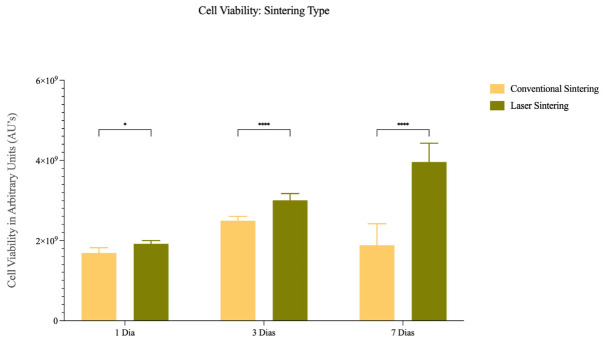
Bar chart comparing the main effects of the hydroxyapatite (HA) sintering methods on human gingival fibroblast (HGF) cell viability over a 7-day culture period. Data represent the combined viability values of the 0.25 mm and 0.8 mm patterned groups processed via either conventional sintering (CS) or laser sintering (LS). Cell viability is expressed in arbitrary units (AUs). Data are presented as mean ± standard deviation (SD) from three independent experiments (*n* = 15 per group). Statistical significance: * *p* < 0.05, **** *p* < 0.001.

When the two types of sintering were compared, as depicted in Figure 2, conventional sintering demonstrated significant and pronounced lower cell viability compared to laser sintering throughout the 7 days of the assay (*p* < 0.05).

### 3.3. Cell Morphology

**Figure 3:**
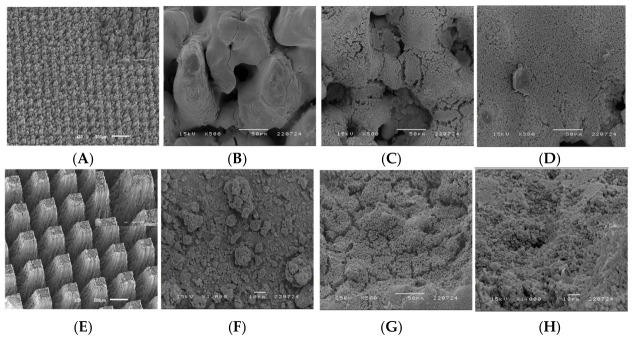
Scanning electron microscopy (SEM) micrographs of laser-textured and hydroxyapatite (HA)-coated titanium surfaces at magnifications of 30× (**A**,**E**), 500× (**B**–**D**,**G**) or 1000× (**F**,**H**). (**A**) Unseeded 0.25 mm laser-textured control (TiT025); (**B**–**D**) human gingival fibroblasts (HGFs) cultured for 24 h on (**B**) TiT025, (**C**) conventionally sintered HA coating (TiT025CS), and (**D**) laser-sintered HA coating (TiT025LS); (**E**) unseeded 0.8 mm laser-textured control (TiT08); (**F**–**H**) HGFs cultured for 24 h on (**F**) TiT08, (**G**) conventionally sintered HA coating (TiT08CS), and (**H**) laser-sintered HA coating (TiT08LS). Micron scale bars and specific magnification factors are embedded directly within each panel.

SEM micrographs obtained from samples after 1 day of fibroblast culture are depicted in Figure 3. Images show adherent cells in all samples after 1 day. However, all CS samples appear to have a lower number of cells than other groups. Fibroblasts present on all samples displayed typical morphology and adequate attachment to the material surface.

### 3.4. Interleukin 1β and Interleukin 10

**Figure 4:**
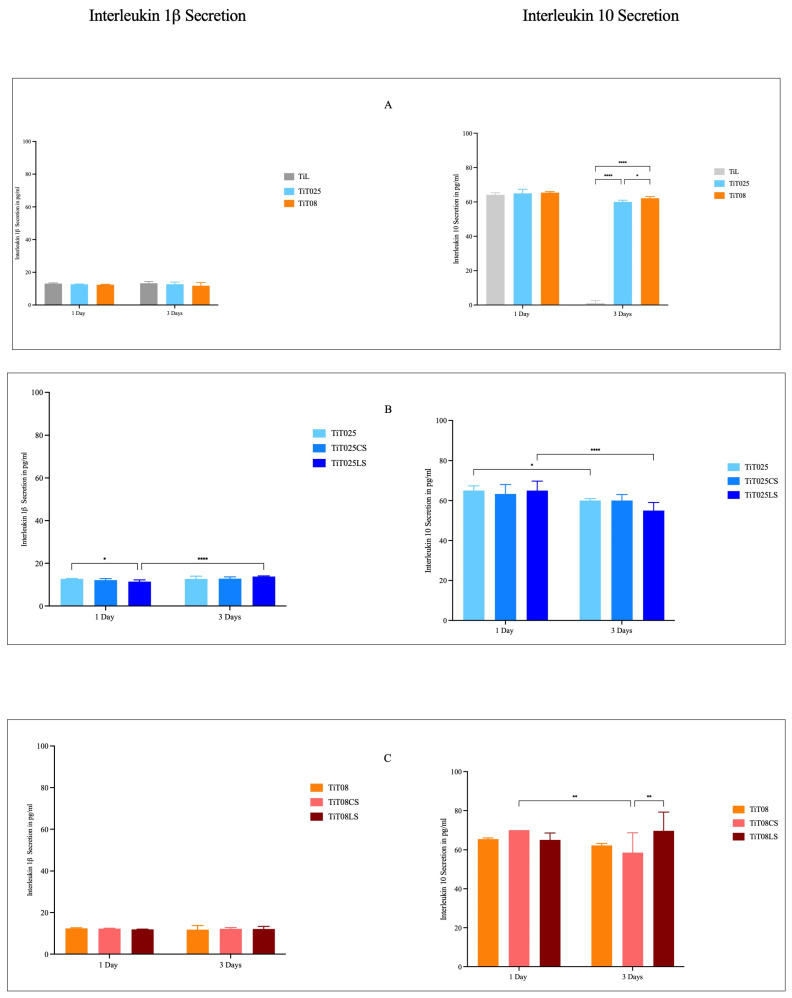
Bar charts depicting the secretion profiles of pro-inflammatory interleukin-1 beta (IL-1b) and anti-inflammatory interleukin-10 (IL-10) from human gingival fibroblasts (HGFs) after 1 and 3 days of culture. (**A**) Cytokine secretion on laser-textured metallic substrates (TiT025 and TiT08) compared to the smooth titanium control (TiL); (**B**) cytokine secretion on the 0.25 mm patterns modified with conventionally sintered (TiT025CS) or laser-sintered (TiT025LS) hydroxyapatite (HA) coatings; (**C**) cytokine secretion on the 0.8 mm patterns modified with conventionally sintered (TiT08CS) or laser-sintered (TiT08LS) HA coatings. Cytokine concentrations are expressed in picograms per milliliter (mL). Data are presented as mean ± standard deviation (SD) from independent experimental replicates (*n* = 6 per group). Statistical significance: * *p* < 0.05, ** *p* < 0.01, **** *p* < 0.001.

IL-1β and IL-10 secretion by fibroblasts was measured in culture media at 1 and 3 days, and the results are presented in Figure 4 as mean cytokine concentrations in pg/mL.

Considering IL-1β secretion, no statistically significant differences were observed between TiL and the textured patterns (TiT025 and TiT08) over the 3-day period (*p* > 0.05). For the 0.25 mm pattern, a reduction in IL-1β secretion was observed at day 1, which was statistically significant for the TiT025LS group (*p* < 0.05). However, after 3 days, no statistically significant differences were detected between groups (*p* > 0.05). Similarly, for the 0.8 mm pattern, no statistically significant differences were found between groups throughout the 3-day study (*p* > 0.05).

Regarding IL-10 secretion, no statistically significant differences were observed at day 1 between TiL and the textured groups (TiT025 and TiT08) (*p* > 0.05). However, at day 3, a marked increase in IL-10 secretion was observed, which was significantly higher in the TiT08 group compared to TiT025 (*p* < 0.05). Within the 0.25 mm pattern, no significant changes in IL-10 secretion were detected at day 1 (*p* > 0.05), whereas a significant reduction was observed after 3 days for both the TiT025 and TiT025LS groups (*p* < 0.05). For the 0.8 mm pattern, no significant differences were found between groups at day 1 (*p* > 0.05). At day 3, IL-10 secretion decreased, with a more pronounced reduction in the TiT08CS group compared to day 1 (*p* < 0.05) and relative to TiT08LS (*p* < 0.05).

**Figure 5:**
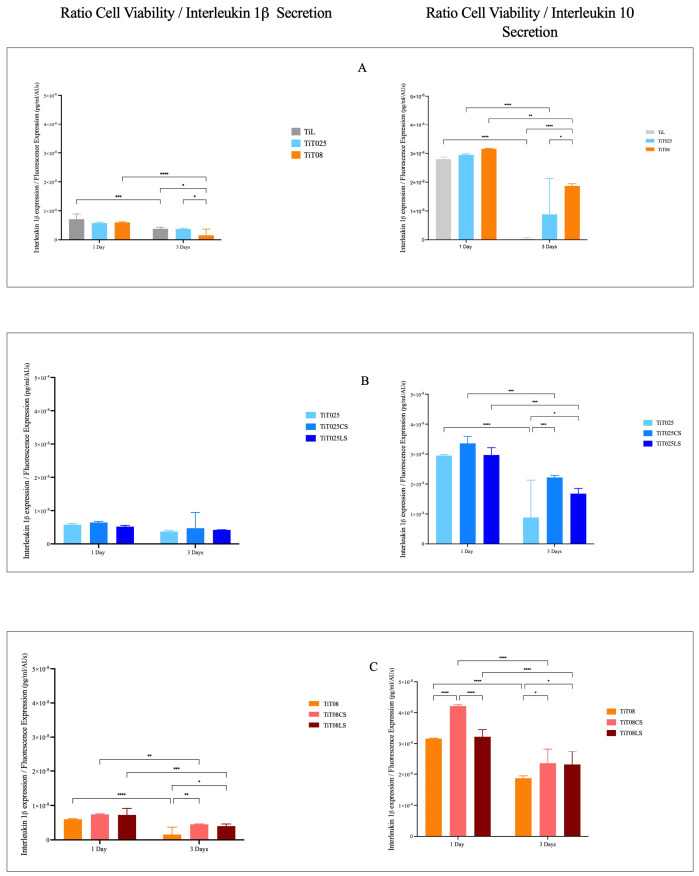
Bar charts depicting the normalized ratio of cytokine secretion to human gingival fibroblast (HGF) cell viability after 1 and 3 days of culture. Panels display the ratio of pro-inflammatory interleukin-1 beta (IL-1b) to viability and anti-inflammatory interleukin-10 (IL-10) to viability, respectively. (**A**) Cytokine/viability ratios on laser-textured metallic substrates (TiT025 and TiT08) compared to the smooth titanium control (TiL); (**B**) cytokine/viability ratios on the 0.25 mm patterns modified with conventionally sintered (TiT025CS) or laser-sintered (TiT025LS) hydroxyapatite (HA) coatings; (**C**) cytokine/viability ratios on the 0.8 mm patterns modified with conventionally sintered (TiT08CS) or laser-sintered (TiT08LS) HA coatings. Ratios are expressed in picograms per milliliter per arbitrary unit (pg/mL/AU). Data are presented as mean ± standard deviation (SD) from independent experimental replicates (*n* = 6 per group). Statistical significance: * *p* < 0.05, ** *p* < 0.01, *** *p* < 0.005, **** *p* < 0.001.

To account for potential variability in cell numbers, an IL-1β/cell viability ratio and IL-10/cell viability ratio were calculated and are presented in Figure 5. Regarding the IL-1β/cell viability ratio, no significant differences were observed at day 1 between TiL and the textured groups (TiT025 and TiT08) (*p* > 0.05). However, at day 3, the TiT08 group showed a significant decrease compared to the other groups (*p* < 0.05). Within the 0.25 mm pattern, no statistically significant differences were detected between groups throughout the 3-day period (*p* > 0.05). For the 0.8 mm pattern, no differences were observed at day 1 (*p* > 0.05); however, at day 3, a marked reduction in the IL-1β/cell viability ratio was observed across all groups compared to day 1 (*p* < 0.05), with a more pronounced decrease, and more statistically significant differences, in TiT08 than in TiT08CS and TiT08LS (*p* < 0.05).

Regarding the IL-10/cell viability ratio, no statistically significant differences were found at day 1 between TiL and the textured groups (TiT025 and TiT08), nor among the TiT025 groups with bioactive addition (TiT025CS and TiT025LS) (*p* > 0.05). Within the 0.8 mm pattern, an increase in the IL-10/cell viability ratio was observed at day 1 in the TiT08CS group compared to TiT08 and TiT08LS (*p* < 0.05). At day 3, a significant reduction in the IL-10/cell viability ratio was observed across all groups when compared to day 1 (*p* < 0.05).

## 4. Discussion

The present study aimed to investigate the biological effects of a novel bioactive coating strategy for titanium implant surfaces. Briefly, we have developed a titanium implant surface with a laser-written checkerboard pattern, incorporating two different surface patterns (0.25 mm and 0.8 mm spacing), followed by the deposition and sintering of hydroxyapatite using either laser or conventional sintering methods. We compared the different gingival fibroblast responses when in contact with these surfaces regarding cell viability, morphology and interaction with the material, as well as the inflammatory secretory profile.

The clinical success of load-bearing orthopedic implants depends on achieving rapid osseointegration while maintaining interface structural integrity during surgical deployment [33,34]. Although medical-grade titanium alloy (Ti6Al4V) provides excellent bulk mechanical strength and corrosion resistance, its inherent surface inertness limits direct biological bonding, which increases the risk of micro-motion [35], localized inflammation [36], and long-term implant loosening. To resolve this limitation, a bioactive hydroxyapatite (Ca10(PO4)6(OH)2) coating is applied to mimic the inorganic mineral phase of native cortical bone, providing an osseoconductive and osseoinductive matrix that directly stimulates the migration, adhesion, and proliferation of cells [22,37,38].

This chemical functionalization alters the interfacial surface energy, inducing strong electrostatic interactions between water molecules and the calcium and phosphate ions during hydration to yield a super-hydrophilic wetting state [39,40]. This extreme wettability accelerates early biochemical reactivity with host proteins and amino acids, while the highly uniform overlayer maximizes chemical affinity with living tissue to accelerate early bone integration [41,42].

Considering the effect of the pattern size, the 0.25 mm pattern creates lower surface roughness than the 0.8 mm pattern. The literature suggests the optimal roughness for fibroblast adhesion is Ra ≈ 0.08–1 µm [14], which is within the surface roughness values of the 0.25 mm pattern (although slightly higher) [43]. This roughness is potentially favorable to the attachment of gingival fibroblasts; however, it may limit proliferation, whereas increased roughness values such as those obtained in 0.8 mm patterns (mean Ra values of 2.7 µm), could potentially be associated with lower biocompatibility [5,44,45]. Scanning electron microscopy images were in line with the viability results, showing fibroblasts with a typical morphology on the titanium surfaces in accordance with viability values. Several studies report the importance of cell morphology and cell viability, indicating a decrease in cell viability when the morphology appears atypical or irregular [46].

When hydroxyapatite was added to the previously textured implant surfaces, regardless of the sintering method, a decrease in cell viability was noted for both patterns across the study. This decrease in viability may be attributed to the difficulty in maintaining stable hydroxyapatite on the surface. The addition of hydroxyapatite to the implant surface presents challenges, particularly in terms of stability, requiring stabilization through sintering techniques [26,27,28]. When evaluating the effect of sintering methods on cell viability, laser sintering demonstrated superior cell viability when compared to conventional sintering techniques. These findings are somewhat contrary to what is typically described in the literature, where the sintering method is not expected to significantly alter biological responses [30].

These results could partially be explained by the concentration of hydroxyapatite used in these previous studies, 0.133 g/mL, which was much lower than in other studies where a 100% concentration was used [47]. Even at relatively low concentrations, hydroxyapatite compounds may exhibit cytotoxic effects on cells, which may explain the observed decrease in cell viability when unstable particles are detached [20]. It would be valuable for future studies to investigate the effects of varying hydroxyapatite concentrations to determine whether the dissociation of hydroxyapatite into the culture medium induces cytotoxicity at specific concentrations.

The interaction between the titanium implant surface and the surrounding soft-tissue fibroblasts dictates the success of the peri-implant biological seal, which is critical for long-term implant stability and the prevention of peri-implantitis [48]. This study investigated the inflammatory response of fibroblasts to various titanium surface modifications by assessing the secretion profiles of the key inflammatory marker IL-1β and the anti-inflammatory cytokine IL-10 over a 3-day period.

Initial analysis at day 1 revealed small statistically significant differences in IL-1β secretion (only for TiT025 vs. TiT025LS, *p* < 0.05) and no statistically significant differences in the secretion of IL-10 across all test groups. This suggests that none of the surface modifications elicited a pronounced, acute inflammatory reaction or cytotoxicity within the first 24 h. This may be regarded as a favorable finding, as severe acute inflammation can impair the healing cascade [49,50,51]. The short-term biocompatibility aligns with numerous studies that confirm the non-cytotoxic nature of commercially pure titanium surfaces on oral soft-tissue cells [52,53]. However, the results at day 3 demonstrated critical differences between study groups, particularly when normalizing cytokine secretion to cell viability, to account for potential differences in cellular proliferation or metabolic activity.

The overall IL-1β profile, especially when measured by the IL-1β/cell viability ratio, serves as an indicator of sustained pro-inflammatory challenge. At day 3, the TIT08 group exhibited a significantly decreased IL-1beta/viability ratio compared to the TIT08CS group (*p* < 0.05). This finding suggests that while both surface treatments are well-tolerated, the specific conventional sintering on the TIT08 substrate did not confer an advantage in reducing the chronic inflammatory signal and, in fact, maintained a significantly higher IL-1 beta burden than the unmodified TIT08 surface [54]. Reduced IL-1β is indicative of a lower chronic inflammatory burden, which is essential given that chronic IL-1β release is a well-known inducer of peri-implant bone loss [55,56]. The role of surface characteristics in modulating pro-inflammatory cytokines is well-established, with some smooth titanium surfaces being linked to M1-like (pro-inflammatory) macrophage activation characterized by elevated IL-1β levels [57,58].

IL-10 is a potent regulatory cytokine that is critical in resolving inflammation and promoting tissue repair and maturation [59]. Analyzing the IL-10/cell viability ratio provides insight into the intrinsic healing and immunoregulatory capacity promoted by each surface treatment. The performance of the surface modifications was most clearly highlighted by the IL-10 data at day 3. The raw data showed the significantly lower IL-10 secretion observed in the TIL group compared to all others, suggesting this unmodified control surface may be the least biocompatible in terms of modulating a favorable anti-inflammatory environment [54,55]. The normalized IL-10 data at day 3 showed that the unmodified TiT025 surface had significantly lower IL-10/viability ratios compared to its modified counterparts TiT025CS and TiT025LS and also compared to TiT08 (*p* < 0.05). This critical distinction demonstrates the high biological impact of CS and LS. These can incorporate HA successfully and transformed the initially poor IL-10 secretion capacity of the TiT025 substrate into a desirable, sustained anti-inflammatory response. Specifically, the increased expression of anti-inflammatory mediators by fibroblasts on modified surfaces supports the literature suggesting that surface treatments designed to optimize topography and chemistry enhance healing functions [60,61,62].

Furthermore, the TIT08CS surface demonstrated a significantly higher IL-10/viability ratio at day 1 compared to the TIT08 and TIT08LS groups. This suggests that the CS treatment on the TIT08 substrate prompts an early, strong anti-inflammatory/regulatory signal, indicating potentially rapid establishment of a pro-healing interface. This early regulatory response is considered highly beneficial for tissue integration, as timely suppression of the initial inflammatory phase prevents chronic inflammation and subsequent fibrous encapsulation of the biomaterial [63,64]. The differences observed across the various CS and LS treatments confirm that both the method of modification and the underlying substrate properties influence the long-term cytokine release. In summary, the transition from day 1 to day 3 cytokine profiles, especially when normalized for cell viability, clearly demonstrates that titanium surface modifications can shift the fibroblast phenotype from a baseline state toward a pro-healing state. The unmodified surfaces (TiT025 and TIL) consistently underperformed in generating the sustained IL-10 response necessary for long-term tissue stability. Conversely, the specific conventional (CS) [65] and laser (LS) treatments applied to the TiT025 substrate proved to be effective strategies, driving a significantly higher and sustained IL-10 response, which is crucial for establishing and maintaining a robust soft-tissue barrier.

While the present study successfully demonstrates that laser-sintered HA coatings yield acceptable early fibroblast responses compared to smooth titanium, certain technical limitations must be acknowledged. First, quantitative roughness characterization was restricted to the metallic substrates, as mechanical contact profilometry can induce micro-damage on thin, brittle ceramic coatings. Future studies utilizing non-contact optical profilometry or atomic force microscopy (AFM) are required to map out sub-micron roughness alterations caused by sintering. Although laser-sintered HA coatings demonstrate acceptable early fibroblast compatibility, this preliminary study lacks comprehensive material characterization regarding coating roughness, mechanical adhesion, and dissolution kinetics. To establish true clinical efficacy from a materials science perspective, future investigations must utilize advanced techniques—such as AFM, scratch testing, XRD, and FTIR—to rigorously evaluate the sub-micron topography, interfacial stability, and structural/chemical integrity of the laser-processed matrix. Future research will be crucial to understand the extent to which other bioactive agents modulate the cellular responses within these implant groups and to explore the role of other cell types, such as osteoblasts, in this process. It will also be essential to investigate the microbiological responses associated with these surfaces, both in isolation and within biofilm environments.

## 5. Conclusions

Although none of the engineered laser-textured patterns or hydroxyapatite coatings outperform the smooth titanium control in absolute cell viability, they consistently support acceptable gingival fibroblast attachment and physiological morphology. Furthermore, the 0.25 mm laser pattern combined with optimized sintering is able to drive a favorable, sustained anti-inflammatory response (up-regulation) necessary for establishing a stable peri-implant soft-tissue barrier.

## Figures and Tables

**Table 1:** Chemical composition of titanium samples [31].

Chemical Composition	Ti	O	Al	V
Wt (%)	78.2	12.7	5.6	3.6

**Table 2:** Nd:YV04 laser specifications. Nd stands for Neodymium, a rare-earth element embedded into the host crystal matrix that acts as the active gain medium responsible for producing the laser emission.

Nd:YV04 Laser	Values
Maximum Output Power [W]	30
Wavelength [32]	1064
Laser Technology	Nd
Repetition Rate Range [kHz]	20
Pulse Width [ns]	10
Spot Size (mm)	0.01
Cooling System	Forced Air-Cooling

**Table 3:** Chemical formula and physicochemical specifications of the hydroxyapatite powder nanoXIM Hap400 (as provided by the manufacturer—Fluidinova, Maia, Portugal).

Parameter	Specification/Value
Chemical Formula	(Ca_10_(PO_4_)_6_(OH)_2_)
Phase Purity	100%
Ca/P Atomic Ratio	1.67–1.68
Particle Size (d_50_)	10.0 ± 2.0 μm
Specific Gravity	0.60 ± 0.10 g/cm^3^
Specific Surface Area	≥100 m^2^/g

**Table 4:** CO_2_ laser specifications [31].

Laser Specifications	Values
Maximum Output Power [W]	50
Wavelength [32]	10,640
Laser Technology	CO_2_
Spot Size (mm)	0.02
Cooling System	None

**Table 5:** Comparative analysis of roughness profiles and wetting properties across samples.

Groups	R_a_ (µm)	CA (°)
TiL	0.2	82.48 ± 2.56
TiT025	1.9	22.22 ± 4.83
TiT025CS	-	6.98 ± 7.14
TiT025LS	-	9.14 ± 6.3
TiT08	2.7	11.25 ± 11.31
TiT08CS	-	0 ± 0
TiT08LS	-	0 ± 0

## Data Availability

The original contributions presented in this study are included in the article. Further inquiries can be directed to the corresponding author.

## References

[1] Pellegrini G., Francetti L., Barbaro B., Del Fabbro M. (2018). Novel surfaces and osseointegration in implant dentistry. J. Investig. Clin. Dent..

[2] Esposito M., Ardebili Y., Worthington H.V. (2014). Interventions for replacing missing teeth: Different types of dental implants. Cochrane Database Syst. Rev..

[3] Albrektsson T., Branemark P.I., Hansson H.A., Lindstrom J. (1981). Osseointegrated titanium implants. Requirements for ensuring a long-lasting, direct bone-to-implant anchorage in man. Acta Orthop. Scand..

[4] Mavrogenis A.F., Dimitriou R., Parvizi J., Babis G.C. (2009). Biology of implant osseointegration. J. Musculoskelet. Neuronal Interact..

[5] Sykaras N., Iacopino A.M., Marker V.A., Triplett R.G., Woody R.D. (2000). Implant materials, designs, and surface topographies: Their effect on osseointegration. A literature review. Int. J. Oral Maxillofac. Implant..

[6] Lee J.W.Y., Bance M.L. (2019). Physiology of Osseointegration. Otolaryngol. Clin. N. Am..

[7] Jung R.E., Pjetursson B.E., Glauser R., Zembic A., Zwahlen M., Lang N.P. (2008). A systematic review of the 5-year survival and complication rates of implant-supported single crowns. Clin. Oral Implant. Res..

[8] Damiati L., Eales M.G., Nobbs A.H., Su B., Tsimbouri P.M., Salmeron-Sanchez M., Dalby M.J. (2018). Impact of surface topography and coating on osteogenesis and bacterial attachment on titanium implants. J. Tissue Eng..

[9] Saini M., Singh Y., Arora P., Arora V., Jain K. (2015). Implant biomaterials: A comprehensive review. World J. Clin. Cases.

[10] Schalock P.C., Menne T., Johansen J.D., Taylor J.S., Maibach H.I., Liden C., Bruze M., Thyssen J.P. (2012). Hypersensitivity reactions to metallic implants—Diagnostic algorithm and suggested patch test series for clinical use. Contact Dermat..

[11] Blazquez-Hinarejos M., Ayuso-Montero R., Jane-Salas E., Lopez-Lopez J. (2017). Influence of surface modified dental implant abutments on connective tissue attachment: A systematic review. Arch. Oral Biol..

[12] Corvino E., Pesce P., Mura R., Marcano E., Canullo L. (2020). Influence of Modified Titanium Abutment Surface on Peri-implant Soft Tissue Behavior: A Systematic Review of In Vitro Studies. Int. J. Oral Maxillofac. Implant..

[13] Meier D., Astasov-Frauenhoffer M., Waltimo T., Zaugg L.K., Rohr N., Zitzmann N.U. (2023). Biofilm formation on metal alloys and coatings, zirconia, and hydroxyapatite as implant materials in vivo. Clin. Oral Implant. Res..

[14] Ponsonnet L., Reybier K., Jaffrezic-Renault N., Comte V., Lagneau C., Lissac M., Martelet C. (2003). Relationship between surface properties (roughness, wettability) of titanium and titanium alloys and cell behaviour. Mater. Sci. Eng. C Mater. Biol. Appl..

[15] Wennerberg A., Albrektsson T. (2009). Effects of titanium surface topography on bone integration: A systematic review. Clin. Oral Implant. Res..

[16] Rupp F., Liang L., Geis-Gerstorfer J., Scheideler L., Huttig F. (2018). Surface characteristics of dental implants: A review. Dent. Mater..

[17] Faria D., Abreu C.S., Buciumeanu M., Dourado N., Carvalho O., Silva F.S., Miranda G. (2018). Ti6Al4V laser surface preparation and functionalization using hydroxyapatite for biomedical applications. J. Biomed. Mater. Res. B Appl. Biomater..

[18] Faria D., Madeira S., Buciumeanu M., Silva F.S., Carvalho O. (2020). Novel laser textured surface designs for improved zirconia implants performance. Mater. Sci. Eng. C Mater. Biol. Appl..

[19] Fernandes B.F., da Cruz M.B., Marques J.F., Madeira S., Carvalho O., Silva F.S., da Mata A., Carames J.M.M. (2020). Laser Nd:YAG patterning enhance human osteoblast behavior on zirconia implants. Lasers Med. Sci..

[20] Cruz M.B.D., Marques J.F., Fernandes B.F., Costa M., Miranda G., Mata A., Carames J.M.M., Silva F.S. (2020). Gingival fibroblasts behavior on bioactive zirconia and titanium dental implant surfaces produced by a functionally graded technique. J. Appl. Oral Sci..

[21] da Cruz M.B., Marques J.F., Fernandes B.F., Pinto P., Madeira S., Carvalho O., Silva F.S., Carames J.M.M., da Mata A. (2022). Laser surface treatment on Yttria-stabilized zirconia dental implants: Influence on cell behavior. J. Biomed. Mater. Res. B Appl. Biomater..

[22] Lukaszewska-Kuska M., Wirstlein P., Majchrowski R., Dorocka-Bobkowska B. (2018). Osteoblastic cell behaviour on modified titanium surfaces. Micron.

[23] Knabe C., Howlett C.R., Klar F., Zreiqat H. (2004). The effect of different titanium and hydroxyapatite-coated dental implant surfaces on phenotypic expression of human bone-derived cells. J. Biomed. Mater. Res. A.

[24] Harimoto K., Yoshida Y., Yoshihara K., Nagaoka N., Matsumoto T., Tagawa Y. (2012). Osteoblast compatibility of materials depends on serum protein absorbability in osteogenesis. Dent. Mater. J..

[25] Nakazawa M., Yamada M., Wakamura M., Egusa H., Sakurai K. (2017). Activation of Osteoblastic Function on Titanium Surface with Titanium-Doped Hydroxyapatite Nanoparticle Coating: An In Vitro Study. Int. J. Oral Maxillofac. Implant..

[26] Eliaz N., Metoki N. (2017). Calcium Phosphate Bioceramics: A Review of Their History, Structure, Properties, Coating Technologies and Biomedical Applications. Materials.

[27] Zhao X. (2011). Introduction to bioactive materials in medicine. Bioactive Materials in Medicine.

[28] Cheng A., Cohen D.J., Kahn A., Clohessy R.M., Sahingur K., Newton J.B., Hyzy S.L., Boyan B.D., Schwartz Z. (2017). Laser Sintered Porous Ti-6Al-4V Implants Stimulate Vertical Bone Growth. Ann. Biomed. Eng..

[29] Lei S., Yan-Feng X., Lu G., Yao W., Fang W., Zhong-Wei G. (2012). The effect of antibacterial ingredients and coating microstructure on the antibacterial properties of plasma sprayed hydroxyapatite coatings. Surf. Coat. Technol..

[30] Mesquita-Guimaraes J., Detsch R., Souza A.C., Henriques B., Silva F.S., Boccaccini A.R., Carvalho O. (2020). Cell adhesion evaluation of laser-sintered HAp and 45S5 bioactive glass coatings on micro-textured zirconia surfaces using MC3T3-E1 osteoblast-like cells. Mater. Sci. Eng. C Mater. Biol. Appl..

[31] Barbosa G.G.M.R. (2022). Surface Functionalization with Antibacterial and Bioactive Compounds Using Hybrid Techniques (Subtractive and Addictive) via Laser for the Improvement of Knee Prostheses Properties. Master’s Thesis.

[32] Jiang P., Zhang Y., Hu R., Shi B., Zhang L., Huang Q., Yang Y., Tang P., Lin C. (2023). Advanced surface engineering of titanium materials for biomedical applications: From static modification to dynamic responsive regulation. Bioact. Mater..

[33] Albrektsson T., Wennerberg A. (2019). On osseointegration in relation to implant surfaces. Clin. Implant Dent. Relat. Res..

[34] Emam S.M., Moussa N. (2024). Signaling pathways of dental implants’ osseointegration: A narrative review on two of the most relevant; NF-κB and Wnt pathways. BDJ Open.

[35] Asensio G., Vázquez-Lasa B., Rojo L. (2019). Achievements in the Topographic Design of Commercial Titanium Dental Implants: Towards Anti-Peri-Implantitis Surfaces. J. Clin. Med..

[36] Bosshardt D.D., Chappuis V., Buser D. (2017). Osseointegration of titanium, titanium alloy and zirconia dental implants: Current knowledge and open questions. Periodontology 2000.

[37] Babel S., Babel S., Peeran S.R., Pratap B. (2025). Nanohydroxyapatite and Bioactive Glass Composites in Bone Regeneration: A Systematic Review. Cureus.

[38] Jin P., Liu L., Chen X., Xi S., Jiang T. (2023). Calcium-to-phosphorus releasing ratio affects osteoinductivity and osteoconductivity of calcium phosphate bioceramics in bone tissue engineering. BioMed. Eng. Online.

[39] Han H.S., Hwang J., Lee S.H., Kim Y., Kim S., Cho Y.-D. (2026). Apatite-coated implant surfaces exhibit superior biological, immunological, and mechanical properties compared to sandblasted acid-etched surfaces. Sci. Rep..

[40] Bernal-Alvarez L.R., Ramirez-Gutierrez C.F., Gomez-Vazquez O.M., Correa-Piña B.A., Zubieta-Otero L.F., Millán-Malo B.M., Rodriguez-Garcia M.E. (2024). Enhancing surface chemistry and wetting behavior of laser-modified Ti–6Al–4V surgical titanium alloy surfaces through wet deposition of biogenic hydroxyapatite. Surf. Coat. Technol..

[41] Nakamura M., Hori N., Ando H., Namba S., Toyama T., Nishimiya N., Yamashita K. (2016). Surface free energy predominates in cell adhesion to hydroxyapatite through wettability. Mater. Sci. Eng. C Mater. Biol. Appl..

[42] Rupp F., Gittens R.A., Scheideler L., Marmur A., Boyan B.D., Schwartz Z., Geis-Gerstorfer J. (2014). A review on the wettability of dental implant surfaces I: Theoretical and experimental aspects. Acta Biomater..

[43] Salazar-Martinez J.D., Forero-Sossa P.A., Henao J., Espinosa-Arbelaez D.G., Poblano-Salas C.A., Trápaga G., Giraldo-Betancur A.L. (2026). Bovine-derived hydroxyapatite coatings produced by flame spraying: Influence of deposition parameters on structural and microstructural characteristics. Surf. Interfaces.

[44] Bartolomeu F., Sampaio M., Carvalho O., Pinto E., Alves N., Gomes J.R., Silva F.S., Miranda G. (2017). Tribological behavior of Ti6Al4V cellular structures produced by Selective Laser Melting. J. Mech. Behav. Biomed. Mater..

[45] Tiainen L., Abreu P., Buciumeanu M., Silva F., Gasik M., Serna Guerrero R., Carvalho O. (2019). Novel laser surface texturing for improved primary stability of titanium implants. J. Mech. Behav. Biomed. Mater..

[46] Rattanapitak R., Thong-ngarm W. (2024). Human gingival fibroblast response on zirconia and titanium implant abutment: A systematic review. J. Prosthodont..

[47] Moisa M., Balasea B.V., Imre M., Vitelaru C., Radulescu R., Duica F., Rus F., Pana I., Muscurel C., Popa A. (2025). Insights into the biocompatibility of human gingival fibroblasts cultured on a hydroxyapatite-coated new Ti-Nb Alloy. New perspectives for dentistry?. Ceram. Int..

[48] Esfahanizadeh N., Motalebi S., Daneshparvar N., Akhoundi N., Bonakdar S. (2016). Morphology, proliferation, and gene expression of gingival fibroblasts on Laser-Lok, titanium, and zirconia surfaces. Lasers Med. Sci..

[49] Dinarello C.A. (2011). Interleukin-1 in the pathogenesis and treatment of inflammatory diseases. Blood.

[50] Galozzi P., Bindoli S., Doria A., Sfriso P. (2021). The revisited role of interleukin-1 alpha and beta in autoimmune and inflammatory disorders and in comorbidities. Autoimmun. Rev..

[51] Murray P.J., Allen J.E., Biswas S.K., Fisher E.A., Gilroy D.W., Goerdt S., Gordon S., Hamilton J.A., Ivashkiv L.B., Lawrence T. (2014). Macrophage activation and polarization: Nomenclature and experimental guidelines. Immunity.

[52] Ellakany P., Alghamdi M.A., Alshehri T., Abdelrahman Z. (2022). Cytotoxicity of Commercially Pure Titanium (cpTi), Silver-Palladium (Ag-Pd), and Nickel-Chromium (Ni-Cr) Alloys Commonly Used in the Fabrication of Dental Prosthetic Restorations. Cureus.

[53] de Souza V.Z., Manfro R., Joly J.C., Elias C.N., Peruzzo D.C., Napimoga M.H., Martinez E.F. (2019). Viability and collagen secretion by fibroblasts on titanium surfaces with different acid-etching protocols. Int. J. Implant Dent..

[54] Rydén L., Molnar D., Esposito M., Johansson A., Suska F., Palmquist A., Thomsen P. (2013). Early inflammatory response in soft tissues induced by thin calcium phosphates. J. Biomed. Mater. Res. A.

[55] Fernandes M.H., Gomes P.D.S. (2016). Bone Cells Dynamics during Peri-Implantitis: A Theoretical Analysis. J. Oral Maxillofac. Res..

[56] De Molon R.S., Vernal R., Oliveira G.E., Steffens J.P., Ervolino E., Theodoro L.H., Van Den Beucken J.J.J.P., Tetradis S. (2026). Inflammatory bone loss and signaling pathways in periodontitis: Mechanistic insights and emerging therapeutic strategies. Bone Res..

[57] Hotchkiss K.M., Clark N.M., Olivares-Navarrete R. (2018). Macrophage response to hydrophilic biomaterials regulates MSC recruitment and T-helper cell populations. Biomaterials.

[58] Refai A.K., Textor M., Brunette D.M., Waterfield J.D. (2004). Effect of titanium surface topography on macrophage activation and secretion of proinflammatory cytokines and chemokines. J. Biomed. Mater. Res. Part A.

[59] Short W.D., Rae M., Lu T., Padon B., Prajapati T.J., Faruk F., Olutoye O.O., Yu L., Bollyky P., Keswani S.G. (2023). Endogenous Interleukin-10 Contributes to Wound Healing and Regulates Tissue Repair. J. Surg. Res..

[60] Jemat A., Ghazali M.J., Razali M., Otsuka Y. (2015). Surface Modifications and Their Effects on Titanium Dental Implants. BioMed Res. Int..

[61] Canullo L., Menini M., Santori G., Rakic M., Sculean A., Pesce P. (2020). Titanium abutment surface modifications and peri-implant tissue behavior: A systematic review and meta-analysis. Clin. Oral Investig..

[62] Shayeb M.A., Elfadil S., Abutayyem H., Shqaidef A., Marrapodi M.M., Cicciù M., Minervini G. (2024). Bioactive surface modifications on dental implants: A systematic review and meta-analysis of osseointegration and longevity. Clin. Oral Investig..

[63] Gomes Y.V.R., Tavares A.A., Barbosa R.C., Tomaz A.F., Sousa W.J.B., Oliveira L.C.C., Silva S.M.L., Fook M.V.L. (2025). Biological responses to biomaterials: A review. Braz. J. Med. Biol. Res..

[64] Browne S., Pandit A. (2015). Biomaterial-Mediated Modification of the Local Inflammatory Environment. Front. Bioeng. Biotechnol..

[65] Berglundh T., Lindhe J., Ericsson I., Marinello C.P., Liljenberg B., Thomsen P. (1991). The soft tissue barrier at implants and teeth. Clin. Oral Implant. Res..

